# Diagnostic Accuracy of circRNAs in Esophageal Cancer: A Meta-Analysis

**DOI:** 10.1155/2019/9673129

**Published:** 2019-08-27

**Authors:** Chen Niu, Lei Zhao, Xudong Guo, Yi Shen, Yi Shao, Fen Liu

**Affiliations:** ^1^Department of Epidemiology and Health Statistics, School of Public Health, Beijing Municipal Key Laboratory of Clinical Epidemiology, Capital Medical University, Beijing 100069, China; ^2^Department of Molecular Physiology and Biophysics, Holden Comprehensive Cancer Center, University of Iowa Carver College of Medicine, Iowa City, 52242 IA, USA

## Abstract

**Purpose:**

Early detection of esophageal cancer is beneficial to the survival and prognosis of patients. Circular RNAs (circRNAs) have been shown to be a potential biomarker for cancer, which can be used for the diagnosis of esophageal cancer. However, the roles of circRNAs in the diagnosis of esophageal cancer has been controversial. The present study, therefore, is aimed at determining the diagnostic accuracy of circRNAs in esophageal cancer.

**Methods:**

Relevant researches were searched from PubMed, Embase, Cochrane Library, OVID, and ISI Web of Science online databases up to March 11, 2019. The estimation of diagnostic indicators, threshold effect, and publication bias were measured by a bivariate binomial mixed model, the Spearman correlation, and Deeks' funnel plot asymmetry test, respectively.

**Results:**

Five studies from 4 articles were included in this meta-analysis. The pooled sensitivity, specificity, overall positive likelihood ratio (PLR), negative likelihood ratio (NLR), diagnostic odds ratio (DOR), and area under the receiver operating characteristics curve (AUC) were 0.79 (95% CI: 0.69-0.87), 0.85 (95% CI: 0.68-0.94), 5.27 (95% CI: 2.46-11.32), 0.24 (95% CI: 0.16-0.36), 21.66 (95% CI: 9.33-50.30), and 0.88 (95% CI: 0.84-0.90), respectively. The Spearman correlation coefficient was 0.60 (*P* = 0.285). The *P* value of Deeks' funnel plot was 0.81.

**Conclusion:**

The above-mentioned results suggested that circRNAs possess a relatively higher diagnostic performance in distinguishing esophageal cancer patients from healthy individuals. Therefore, they may serve as potential clinical biomarkers for esophageal cancer diagnosis.

## 1. Introduction

Esophageal cancer ranks seventh in terms of incidence and sixth in mortality overall, with the highest incidence rate in Eastern Asia [1]. Esophageal squamous cell carcinoma (ESCC) and adenocarcinoma (EADC) are the most common histologic subtypes that have different etiologies [2]. EADC was considered the most common type of esophageal cancer in high-income countries, and its main risk factors were excess body weight and gastroesophageal reflux disease (GERD) ([3]). High incidence of ESCC has been reported in Southeastern and Central Asia [2]. In China, ESCC was the major type of esophageal cancer, which was caused by multiple etiologies including diet behaviors, lifestyles, and genetic factors [4]. Moreover, most esophageal cancer patients have a poor prognosis, due to the late stage at diagnosis. At present, endoscopy and pathological biopsy are the gold standards for the diagnosis of esophageal cancer [5]. However, it is impossible to avoid damage to the patients during the operation of endoscopy. Furthermore, traditional tumor markers have limited diagnostic performance. Thus, many researchers were exploring a more accurate and less invasive screening tool to allow the early detection of esophageal cancer.

Circular RNA (circRNA), a novel class of endogenous noncoding RNA, was first discovered in RNA viruses, once considered a kind of abnormal splicing products of RNAs [6, 7]. After back splicing of exons, introns, or both, circRNAs possessing a covalently closed continuous loop with neither 5′-3′ polarity nor a polyadenylated tail were formed [8, 9]. The closed continuous loop structure of circRNAs can prevent degradation from RNA exonuclease or RNase R and is more stable than that of linear RNAs [10]. Besides, more than eighty percent circRNAs are overlapping with protein-coding regions and the copy number of circRNAs is also almost ten times higher than that of the associated linear RNAs, which indicated that circRNAs could play a major role or as a sort of offbeat biomarker in diseases [11–14]. According to published articles, there are several main functions of circRNAs in mammalian cells, which comprise affecting microRNAs (miRNAs) functioning as miRNA sponges, modulating alternative splicing and transcription, regulating cell cycle, and being translated into protein by N6-methyladenosine (m6A) modification [14, 15]. Parts of circRNAs have been involved in regulating multiple cellular processes of cancer, such as proliferation, differentiation, and apoptosis [16, 17]. Recent studies had shown that circRNAs are dysregulated in esophageal cancer, and some upregulated circRNAs can promote cell proliferation [18, 19]. In addition, circRNAs are related to diagnosing ESCC patients according to several reports. However, these references have reported inconsistent results. Fan et al. found that hsa_circ_0001946 was lowly expressed in the plasma of ESCC patients and the sensitivity and specificity were 92% and 80%, respectively [20]. Nonetheless, Wang et al. reported circ-TTC17 with a sensitivity of 73% and a specificity of 88% for the detection of ESCC [21]. Thus, the purpose of this systematic review and meta-analysis is to explore the overall diagnostic values of circRNAs as promising biomarkers for esophageal cancer detection.

## 2. Materials and Methods

### 2.1. Search Strategy

This study was performed in accordance with the preferred reporting items for systematic reviews and meta-analyses (PRISMA) checklist ([Supplementary-material supplementary-material-1]) [22]. An electronic search was carried out in PubMed, Embase, Cochrane Library, OVID (All resource), and ISI Web of Science databases up to March 11, 2019, with English language only. The literature search process was conducted by Chen Niu and Xudong Guo. The mesh terms or keywords used for literature retrieval were as follows: (“Esophageal Neoplasms” OR ((“esophageal∗” OR “esophagus∗”) AND (“cancer∗” OR “carcinoma∗” OR “tumor∗” OR “neoplasm∗”))) AND (“circular RNA∗” OR “circRNA∗” OR “circ∗”) AND (“diagnosis” OR “diagnose∗” OR “biomarker”).

### 2.2. Study Selection

The included articles must meet the following criteria: (1) the studies assessed the diagnostic accuracy of circRNAs in esophageal cancer; (2) sufficient data were provided or could be calculated in articles, including true positive (TP), false positive (FP), false negative (FN), and true negative (TN). The exclusion criteria are as follows: (1) duplicate publications and (2) reviews, letters, conference abstracts, poster, and case reports. Two independent researchers (Yi Shen and Yi Shao) assessed studies according to the above criteria. If there were disagreements, they were resolved through discussion with the third researcher (Fen Liu).

### 2.3. Data Extraction and Quality Assessment

Two reviewers (Chen Niu and Yi Shen) independently extracted the following data from eligible studies: the first author's last name, year of publication, country, cancer type, circRNA expression signature, sample size, the area under the receiver operating characteristic (ROC) curve (AUC), sensitivity, and specificity, as well as TP, FP, FN, and TN. The value of TP, FP, FN, and TN could be estimated in line with sensitivity, specificity, sample size, or AUC if these parameters were not supplied in included articles.

Quality Assessment of Diagnostic Accuracy Studies (QUADAS-2), a revised tool, was used to evaluate the quality of the included studies, which consist of four domains including patient selection, index test, reference standard, and flow and timing [23]. The accurate quality score of each article relies on the 14 items from four domains, and any item was appraised as +1 (yes) or 0 (unclear or no). Quality assessment of the included study was completed by two independent investigators (Chen Niu and Xudong Guo).

### 2.4. Statistical Analyses

STATA version 13.0 (StataCorp, College Station, TX, USA) and Meta-DiSc version 1.4 were employed to analyze all data. The pooled sensitivity, specificity, positive likelihood ratio (PLR), negative likelihood ratio (NLR), diagnostic odds ratio (DOR), summary receiver operator characteristic (sROC) curve, and area under the sROC curve (AUC) with 95% confidence intervals (CIs) were calculated to assess the diagnostic performance of circRNA for esophageal patients. The estimation of diagnostic parameters was summarized using the bivariate binomial mixed model. The Spearman correlation coefficient of logarithm sensitivity and 1 − specificity was calculated to detect the threshold effect [24]. Chi-squared and *I*^2^ statistics were used to assess the nonthreshold effect. A value of *P* < 0.1 or *I*^2^ > 50% was considered significant heterogeneity caused by a nonthreshold effect. Subgroup analyses and metaregression were performed to explore the potential heterogeneity among the included studies. In addition, Deeks' funnel plot was used to investigate the publication bias. All *P* values were two-sided, and the statistical significance was defined as *P* < 0.05.

## 3. Results

### 3.1. Characteristics and Quality Assessments of Included Studies

**Figure 1:**
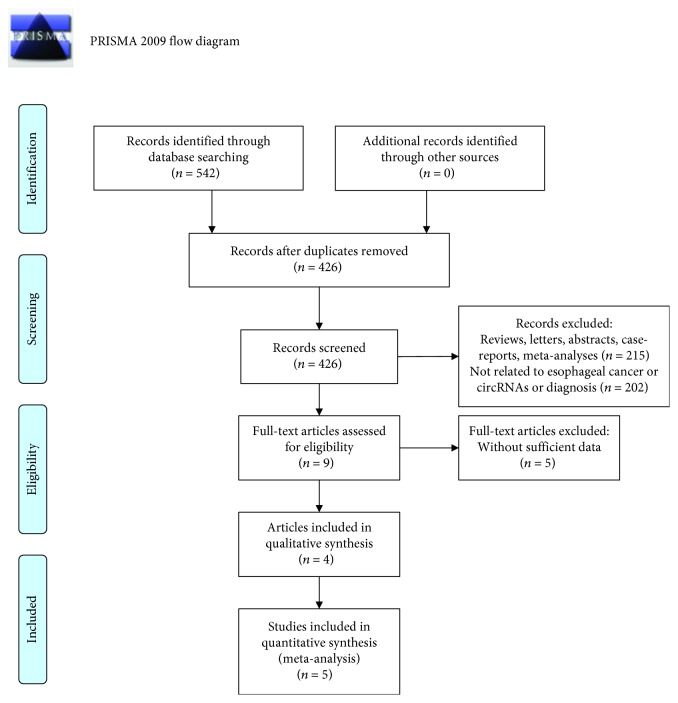
PRISMA 2009 flow diagram in our study.

A total of 542 records were identified from five databases (Figure 1). Four hundred and twenty-six records were remained after removing duplicates. After the title and abstract were reviewed, 9 articles were subjected to further full-text review, of which 5 records were excluded without sufficient data. Finally, 4 articles were included for meta-analysis (Table 1) [20, 21, 25, 26].

If an article contained multiple independent circRNA assays, the test results were treated as separate datasets [20]. As a result, a total of 5 datasets from 4 articles were analyzed in the quantitative synthesis. Overall, 5 types of circRNAs and 275 individuals (147 patients and 128 controls) were included. Among them, the articles of Rong et al., Wang et al., and Zhang et al. provided a single circRNA-based study. The publication of Fan et al. provided two circRNA datasets. All studies used high-throughput human circRNA microarray to screen the dysregulated circRNA expression profiles, and each circRNA expression was measured by quantitative real-time reverse transcription PCR (qRT-PCR). All cases were ESCC patients, and the sample type was plasma. All included studies were conducted in China, which was published from 2018 to 2019 (Table 1).

In addition, QUADAS-2 scores were used to evaluate the quality of the included studies. Overall, all the studies had a score ≥ 6, indicating a moderately high quality (Table 1).

### 3.2. Diagnostic Accuracy Analysis

**Figure 2:**
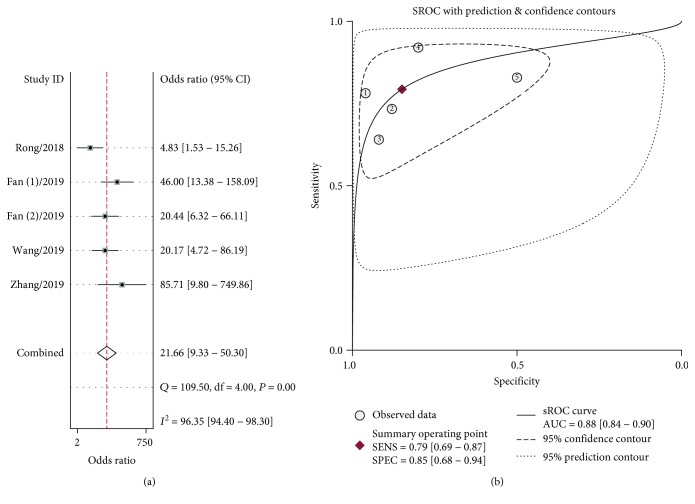
The diagnostic odds ratio (DOR) (a) and the summary receiver operator characteristic (sROC) curve with pooled estimates of sensitivity, specificity, and the area under the (sROC) curve (AUC) (b) on the diagnostic value of circRNAs in esophageal cancer.

Five datasets were finally included in this meta-analysis.  Table 2 summarizes the pooled estimates of diagnostic accuracy and the corresponding 95% CI of included circRNAs. The pooled sensitivity and specificity were 0.79 (95% CI: 0.69-0.87) (Supplementary [Supplementary-material supplementary-material-1]a) and 0.85 (95% CI: 0.68-0.94), respectively (Supplementary [Supplementary-material supplementary-material-1]b). The overall DOR was 21.66 (95% CI: 9.33-50.30) (Figure 2(a)), and the AUC was 0.88 (95% CI: 0.84-0.90) (Figure 2(b)). Furthermore, the summarized PLR and NLR were 5.27 (95% CI: 2.46-11.32) and 0.24 (95% CI: 0.16-0.36), respectively (Supplementary [Supplementary-material supplementary-material-1]c and [Supplementary-material supplementary-material-1]d).

### 3.3. Test of Heterogeneity and Subgroup Analysis

The threshold effect, a potential source of heterogeneity, can be determined by the Spearman correlation coefficient. The Meta-DiSc software was used to analyze the threshold effect of the present meta-analysis, and the Spearman correlation coefficient was 0.60 (*P* = 0.285), indicating that the threshold effect was not the main source of heterogeneity in this study.

In addition, the nonthreshold effect may also contribute to heterogeneity, which cannot be avoided in meta-analysis. Based on the *I*^2^ and *P* value of the overall sensitivity (*I*^2^ = 67.82%, *P* = 0.01), specificity (*I*^2^ = 85.20%, *P* < 0.001), PLR (*I*^2^ = 73.31%, *P* < 0.001), NLR (*I*^2^ = 55.41%, *P* = 0.06), and DOR (*I*^2^ = 96.35%, *P* < 0.001), the results indicated that significant heterogeneity was observed among included studies. Therefore, we applied the bivariate binomial mixed model to summarize the pooled estimates.

To determine the source of heterogeneity, we carried out a metaregression analysis to examine the effects of sample size (*n* ≥ 70/*n* < 70) and circRNA expression (upregulated/downregulated). Although the metaregression results were negative in these factors (Supplementary [Supplementary-material supplementary-material-1]), we still conducted subgroup analyses based on the sample size and circRNA expression pattern to further explore the potential diagnostic value of circRNAs included in this study (Table 2). First, we performed the subgroup analysis based on the sample size (the sample size of 2 studies was greater than 70, and the other 2 studies had a sample size less than 70). Except for the sensitivity, the diagnostic accuracy of circRNAs in the subgroup of *n* ≥ 70 (sensitivity, 0.78; specificity, 0.86; PLR, 5.29; NLR, 0.21; DOR, 30.04) had nonsignificantly better ESCC diagnostic accuracy than that of the subgroup of *n* < 70 (sensitivity, 0.78; specificity, 0.77; PLR, 5.00; NLR, 0.28; DOR, 16.59) (Table 2). Regarding the expression pattern, the downregulated circRNAs showed nonsignificantly higher diagnostic accuracy compared with the upregulated circRNAs, with specificity increasing from 0.68 to 0.88, PLR increasing from 2.94 to 6.42, NLR decreasing from 0.32 to 0.23, and DOR increasing from 9.19 to 34.55. However, the sensitivity (0.78) was similar (Table 2).

### 3.4. Sensitivity Analysis and Publication Bias

According to the result of sensitivity analysis, there was no serious effect on this meta-analysis by each individual induced study (Supplementary [Supplementary-material supplementary-material-1]). Finally, publication bias was measured by Deeks' funnel plot asymmetry test, and the result suggested no publication bias present in the end (*P* = 0.81) (Supplementary [Supplementary-material supplementary-material-1]).

## 4. Discussion

To the best of our knowledge, this article is the first systematic review and meta-analysis to evaluate the diagnostic capability of circRNAs in discriminating esophageal cancer. Our meta-analysis has shown that, as a diagnostic biomarker, plasma circRNAs had a relatively high diagnostic accuracy and achieved a combined AUC of 0.88 (Figure 2(b)) with 79% pooled sensitivity (Supplementary [Supplementary-material supplementary-material-1]a) and 85% specificity (Supplementary [Supplementary-material supplementary-material-1]b) in discriminating ESCC patients (Table 2). The pooled DOR in our analysis was 21.66, which is a diagnostic performance index incorporating the advantages of sensitivity and specificity; the higher value of DOR, the more power of discrimination for patients and healthy controls. We also found that the overall PLR and NLR were 5.27 and 0.24, respectively. The PLR refers to the probability of having esophageal cancer in a person with a positive result about 5-fold higher compared to the person without the disease. The NLP means that the probability of a patient having ESCC is 24% if the circRNA assay shows a negative result. Together, these results indicated that plasma circRNAs are clearly able to discriminate ESCC cases from healthy controls.

Heterogeneity, a potential problem of meta-analysis, affected the validity of results in a systematic review and needed to quantify. The threshold effect is one of the primary causes of heterogeneity among diagnostic studies. In the present analysis, we did not find significant heterogeneity caused by the threshold effect. Furthermore, we conducted a meta-regression to evaluate the effect of sample size (*n* ≥ 70 or *n* < 70) and circRNA expression pattern (upregulated or downregulated). None were found to be the sources of heterogeneity for this study (Supplementary [Supplementary-material supplementary-material-1]). In addition, sensitivity analysis (Supplementary [Supplementary-material supplementary-material-1]) and Deeks' funnel plot asymmetry test (Supplementary [Supplementary-material supplementary-material-1]) were conducted to detect the outliers and publication bias with the results confirming the robustness of our meta-analysis results.

There are five circRNAs that were identified in this meta-analysis. Among them, the expression levels of circ-DLG1 and circ-TTC17 were upregulated, yet hsa_circ_0001946, hsa_circ_0062459, and circ-SMAD7 were downregulated in the ESCC plasma, as the previous studies demonstrated that the expression levels of circ-DLG1 and circ-TTC17 were aberrantly increased in the ESCC tissue and esophageal cancer cells. The expressions of hsa_circ_0001946 and circ-SMAD7 were downregulated in esophageal cancer cell lines. In addition, it has been suggested that both circ-TTC17 and hsa_circ_0001946 may serve as promising prognostic indicators. Furthermore, circ-TTC17 and circ-DLG1 might influence the function of ESCC through circRNA-miRNA-mRNA network [21, 25]. These findings provide important evidence for the circRNAs which play important roles during the carcinogenesis of the esophagus.

This meta-analysis was performed in compliance with the PRISMA guideline, using multiple search strategies and by independent reviewers. We have carefully defined the inclusion and exclusion criteria so that all the studies included in our meta-analysis had acceptable quality and the cases and controls were collated from all included studies. We have used appropriate statistical methods and interpretation through which the statistical power was significantly increased. However, the limitations of this study still need to be declared. First, the sample size of involved participants in the 4 eligible articles is quite small; thus, larger cohorts are needed to confirm the conclusions in further researches. Second, the subgroup analysis of an individual circRNA biomarker could not be conducted due to restricted information and a limited number of articles. Lastly, as shown in Table 1, all included the studies were from the Chinese population; therefore, further studies on Caucasian, African, and other populations are needed.

## 5. Conclusion

Our meta-analysis illustrated plasma circRNAs maintaining acceptable sensitivity and specificity for distinguishing ESCC patients from healthy individuals, which indicated that plasma circRNAs could be a potential innovative biomarker for ESCC diagnosis. Next, more different ethnic populations, larger sample size, and prospective research are desired to substantiate the diagnostic performance of circRNAs.

## Figures and Tables

**Table 1:** Main characteristics of the included studies in this meta-analysis.

References	Year	Country	Case/control	circRNAs	Regulation	Cancer type	Specimen	Sensitivity (%)	Specificity (%)	AUC	TP	FP	FN	TN	QUADAS-2
Rong et al.	2018	China	35/28	circ-DLG1	Upregulated	ESCC	Plasma	83.0	50.0	0.648	29	14	6	14	7
Fan et al.	2019	China	50/50	hsa_circ_0001946	Downregulated	ESCC	Plasma	92.0	80.0	0.894	46	10	4	40	8
				hsa_circ_0062459	Downregulated	ESCC	Plasma	64.0	92.0	0.836	32	4	18	46	
Wang et al.	2019	China	30/25	circ-TTC17	Upregulated	ESCC	Plasma	73.3	88.0	0.820	22	3	8	22	7
Zhang et al.	2019	China	32/25	circ-SMAD7	Downregulated	ESCC	Plasma	78.0	96.0	0.859	25	1	7	24	6

ESCC: esophageal squamous cell carcinoma; AUC: area under the receiver operating characteristic curve; TP: true positive; FP: false positive; FN: false negative; TN: true negative.

**Table 2:** Detailed information of overall meta-analysis and subgroup analyses.

Analysis	No. of studies	Sensitivity (95% CI)	Specificity (95% CI)	PLR (95% CI)	NLR (95% CI)	DOR (95% CI)	TP	FP	FN	TN
Overall	5	0.79 (0.69-0.87)	0.85 (0.68-0.94)	5.27 (2.46-11.32)	0.24 (0.16-0.36)	21.66 (9.33-50.30)	154	32	43	146
Sample size										
≥70	2	0.78 (0.69-0.86)	0.86 (0.78-0.92)	5.29 (3.26-8.59)	0.21 (0.05-0.93)	30.04 (12.83-70.33)	78	14	22	86
<70	3	0.78 (0.69-0.86)	0.77 (0.66-0.68)	5.00 (0.89-28.03)	0.28 (0.19-0.42)	16.59 (3.49-78.98)	76	18	21	60
circRNA expression										
Upregulated	2	0.78 (0.67-0.88)	0.68 (0.54-0.80)	2.94 (0.70-12.41)	0.32 (0.19-0.52)	9.19 (2.28-37.04)	51	17	14	36
Downregulated	3	0.78 (0.70-0.85)	0.88 (0.81-0.93)	6.42 (3.28-12.59)	0.23 (0.10-0.50)	34.55 (15.65-76.27)	103	15	29	110

95% CI: 95% confidence interval; PLR: positive likelihood ratio; NLR: negative likelihood ratio; DOR: diagnostic odds ratio; TP: true positive; FP: false positive; FN: false negative; TN: true negative.

## Data Availability

The data supporting this meta-analysis are from previously reported studies and datasets, which have been cited. The processed data are available in the article and supplementary material.

## Supplementary Materials

Supplementary 1 Supplementary Table 1: detailed information on metaregression. Supplementary Figure 1: forest plots of pooled sensitivity (a), specificity (b), positive likelihood ratio (PLR) (c), and negative likelihood ratio (NLR) (d) of circRNAs for the diagnosis of esophageal cancer. Supplementary Figure 2: sensitivity analysis of the included studies. Supplementary Figure 3: Deeks' funnel plot of the included studies.

Supplementary 2 Supplementary PRISMA 2009 checklist.

## References

[1] Bray F., Ferlay J., Soerjomataram I., Siegel R. L., Torre L. A., Jemal A. (2018). Global cancer statistics 2018: GLOBOCAN estimates of incidence and mortality worldwide for 36 cancers in 185 countries. *CA: A Cancer Journal for Clinicians*.

[2] Arnold M., Soerjomataram I., Ferlay J., Forman D. (2015). Global incidence of oesophageal cancer by histological subtype in 2012. *Gut*.

[3] Blot W. J., Tarone R. E., Thun M. J., Linet M. S., Cerhan J. R., Haiman C. A., Schottenfeld D. (2018). Esophageal cancer. *Cancer Epidemiology and Prevention*.

[4] Abnet C. C., Arnold M., Wei W. Q. (2018). Epidemiology of esophageal squamous cell carcinoma. *Gastroenterology*.

[5] Pennathur A., Farkas A., Krasinskas A. M. (2009). Esophagectomy for T1 esophageal cancer: outcomes in 100 patients and implications for endoscopic therapy. *The Annals of Thoracic Surgery*.

[6] Cocquerelle C., Mascrez B., Hetuin D., Bailleul B. (1993). Mis-splicing yields circular RNA molecules. *The FASEB Journal*.

[7] Sanger H. L., Klotz G., Riesner D., Gross H. J., Kleinschmidt A. K. (1976). Viroids are single-stranded covalently closed circular RNA molecules existing as highly base-paired rod-like structures. *Proceedings of the National Academy of Sciences of the United States of America*.

[8] Barrett S. P., Salzman J. (2016). Circular RNAs: analysis, expression and potential functions. *Development*.

[9] Greene J., Baird A. M., Brady L. (2017). Circular RNAs: biogenesis, function and role in human diseases. *Frontiers in Molecular Biosciences*.

[10] Jeck W. R., Sorrentino J. A., Wang K. (2013). Circular RNAs are abundant, conserved, and associated with ALU repeats. *RNA*.

[11] Guo J. U., Agarwal V., Guo H., Bartel D. P. (2014). Expanded identification and characterization of mammalian circular RNAs. *Genome Biology*.

[12] Han B., Chao J., Yao H. (2018). Circular RNA and its mechanisms in disease: from the bench to the clinic. *Pharmacology & Therapeutics*.

[13] Memczak S., Jens M., Elefsinioti A. (2013). Circular RNAs are a large class of animal RNAs with regulatory potency. *Nature*.

[14] Xia W., Qiu M., Chen R. (2016). Circular RNA has_circ_0067934 is upregulated in esophageal squamous cell carcinoma and promoted proliferation. *Scientific Reports*.

[15] Yang Y., Fan X., Mao M. (2017). Extensive translation of circular RNAs driven by N^6^-methyladenosine. *Cell Research*.

[16] Shang Q., Yang Z., Jia R., Ge S. (2019). The novel roles of circRNAs in human cancer. *Molecular Cancer*.

[17] Zhong Y., du Y., Yang X. (2018). Circular RNAs function as ceRNAs to regulate and control human cancer progression. *Molecular Cancer*.

[18] Huang H., Wei L., Qin T., Yang N., Li Z., Xu Z. (2018). Circular RNA ciRS-7 triggers the migration and invasion of esophageal squamous cell carcinoma via miR-7/KLF4 and NF-*κ*B signals. *Cancer Biology & Therapy*.

[19] Song H., Xu D., Shi P. (2019). Upregulated circ RNA hsa_circ_0000337 promotes cell proliferation, migration, and invasion of esophageal squamous cell carcinoma. *Cancer Management and Research*.

[20] Fan L., Cao Q., Liu J., Zhang J., Li B. (2019). Circular RNA profiling and its potential for esophageal squamous cell cancer diagnosis and prognosis. *Molecular Cancer*.

[21] Wang Q., Zhang Q., Sun H. (2019). Circ-TTC17 promotes proliferation and migration of esophageal squamous cell carcinoma. *Digestive Diseases and Sciences*.

[22] Moher D., Shamseer L., Clarke M. (2015). Preferred reporting items for systematic review and meta-analysis protocols (PRISMA-P) 2015 statement. *Systematic Reviews*.

[23] Whiting P. F., Rutjes A. W., Westwood M. E. (2011). QUADAS-2: a revised tool for the quality assessment of diagnostic accuracy studies. *Annals of Internal Medicine*.

[24] Zamora J., Abraira V., Muriel A., Khan K., Coomarasamy A. (2006). Meta-DiSc: a software for meta-analysis of test accuracy data. *BMC Medical Research Methodology*.

[25] Rong J., Wang Q., Zhang Y. (2018). Circ-DLG1 promotes the proliferation of esophageal squamous cell carcinoma. *OncoTargets and therapy*.

[26] Zhang Y., Wang Q., Zhu D., Rong J., Shi W., Cao X. (2019). Up-regulation of circ-SMAD7 inhibits tumor proliferation and migration in esophageal squamous cell carcinoma. *Biomedicine & Pharmacotherapy*.

