# Supplementary material for: Diagnostic Accuracy of circRNAs in Esophageal Cancer: A Meta-Analysis
Source: Dis Markers. 2019 Aug 27;2019:9673129. doi: 10.1155/2019/9673129 (PMC6732612; doi:10.1155/2019/9673129)
Supplement: Supplementary 1 — Supplementary Table 1: detailed information on metaregression. Supplementary Figure 1: forest plots of pooled sensitivity (a), specificity (b), positive likelihood ratio (PLR) (c), and negative likelihood ratio (NLR) (d) of circRNAs for the diagnosis of esophageal cancer. Supplementary Figure 2: sensitivity analysis of the included studies. Supplementary Figure 3: Deeks' funnel plot of the included studies. [file 9673129.f1.doc]

Supplementary Table 1: Detailed information on meta-regression.

| Variables | Coeff. | Std. Err. | *P*-value | RDOR | 95% Cl |
| --- | --- | --- | --- | --- | --- |
| Sample size | 0.567 | 1.0945 | 0.6561 | 1.76 | 0.02-195.57 |
| Expression | -1.253 | 0.9418 | 0.3148 | 0.29 | 0.00-16.43 |

95% CI: 95% confidence interval; Sample size: ≥70 or <70; Expression: up-regulated or down-regulated.


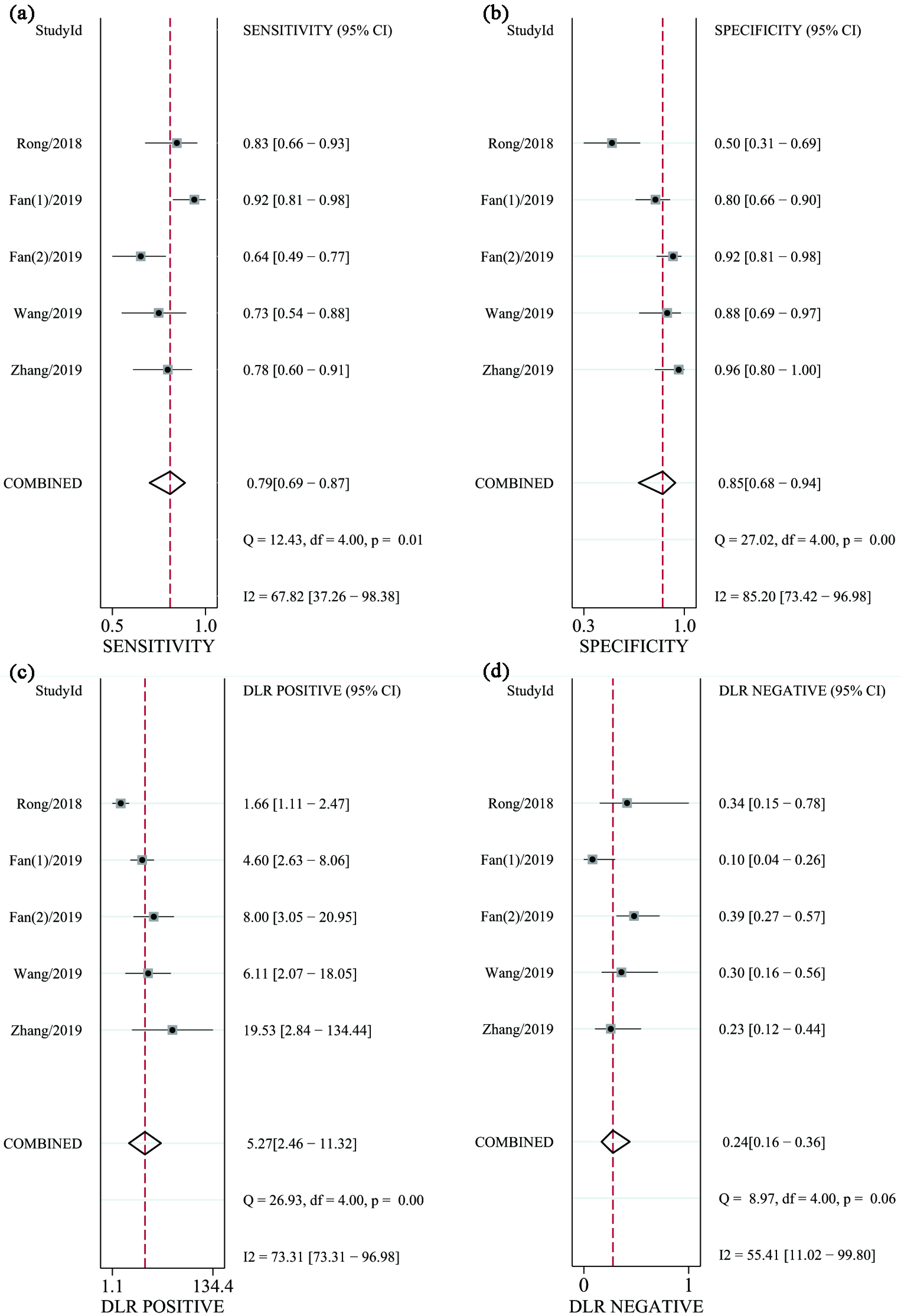


Supplementary Figure 1: Forest plots of pooled sensitivity (a), specificity (b), positive likelihood ratio (PLR) (c) and negative likelihood ratio (NLR) (d) of circRNAs for the diagnosis of esophageal cancer.


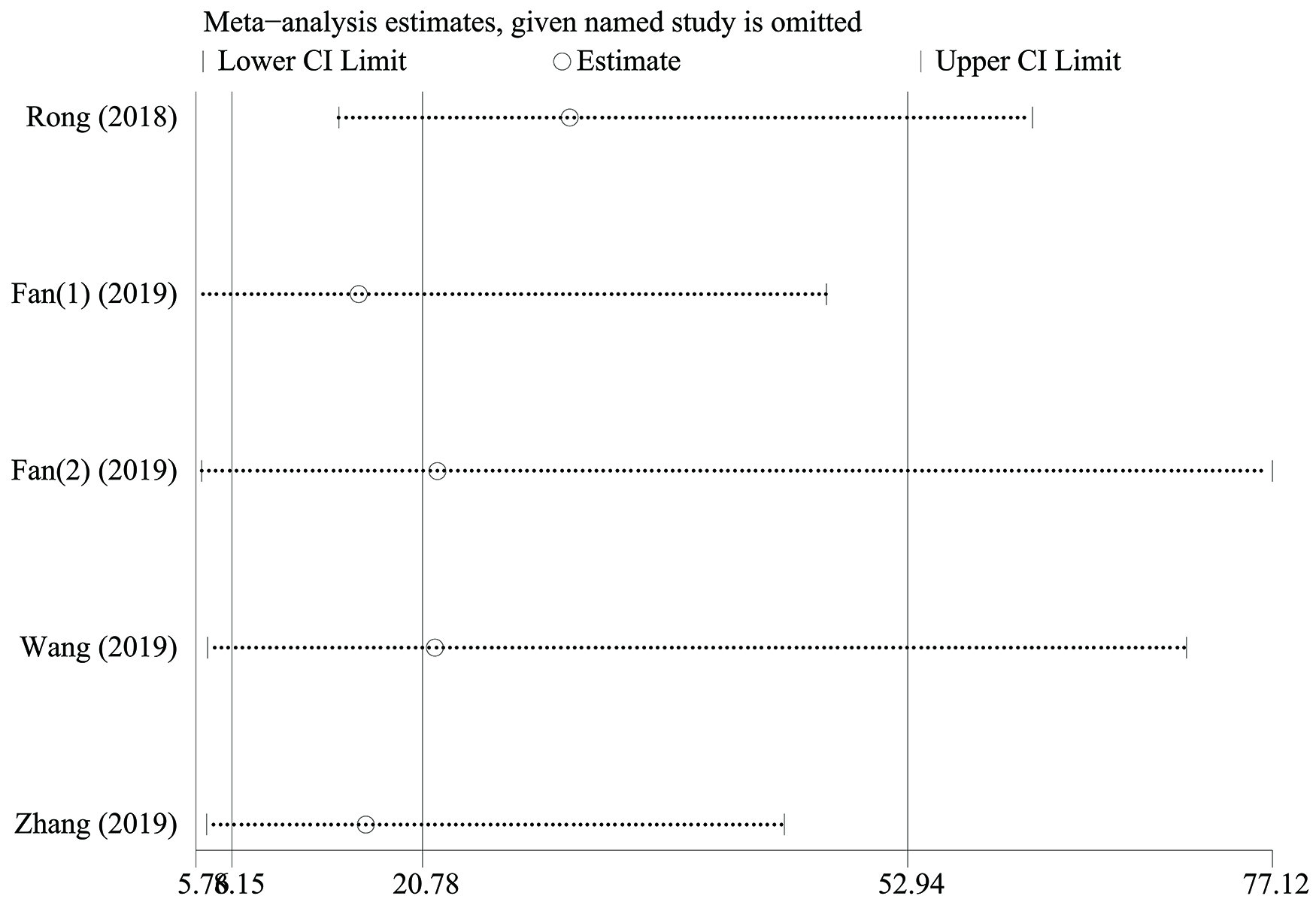


Supplementary Figure 2: sensitivity analysis of the included studies.


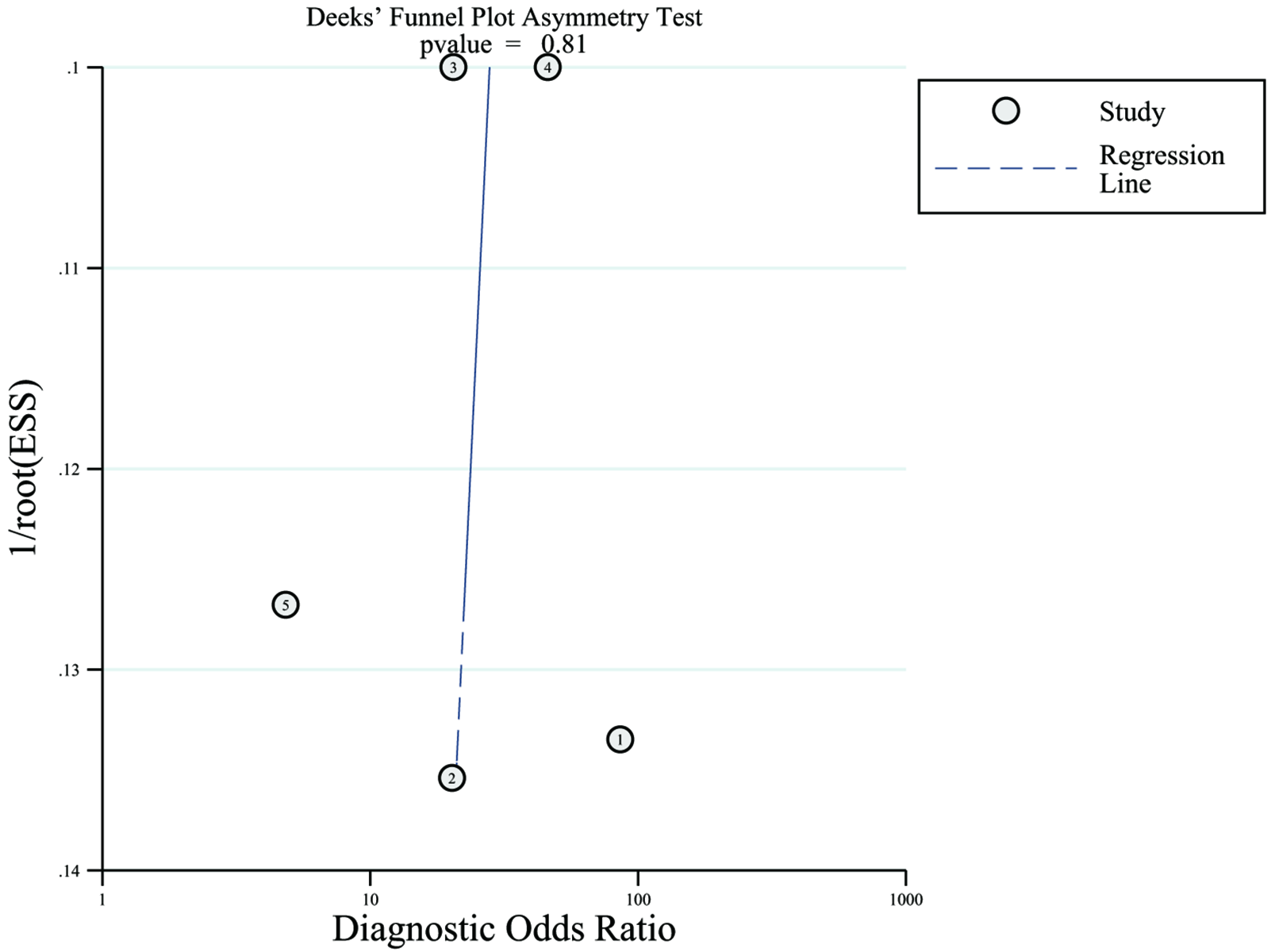


Supplementary Figure 3: Deek’ s funnel plot of the included studies.
